# Examining how fiscal policies influence innovation in TCM enterprises: the role of R&D investment and executives with pharmaceutical backgrounds

**DOI:** 10.3389/fpubh.2025.1531622

**Published:** 2025-02-26

**Authors:** Dan Guo, Liwen Qi, Xiaoting Song

**Affiliations:** Shanghai International College of Intellectual Property, Tongji University, Shanghai, China

**Keywords:** innovation subsidies, tax incentives, executives with pharmaceutical backgrounds, traditional Chinese medicine, enterprise innovation

## Abstract

**Introduction:**

Innovation is crucial to realize the modernization and industrialization of traditional Chinese medicine (TCM), so its incentive methods and influence mechanisms are worth exploring. Based on externality theory and imprinting theory, this paper demonstrates the significance of external support and internal resources in the innovation of TCM enterprises.

**Methods:**

This study adopts a sample of listed TCM enterprises in China during 2007–2023 to examine the impacts and differences between innovation subsidies and tax incentives on TCM enterprise innovation. Innovation in TCM enterprises is deconstructed into five dimensions, including innovation quantity (InNum), innovation quality (InQua), substantive innovation (SubIn), strategic innovation (StrIn), and inheritance innovation (InhIn).

**Results:**

It is found that the incentive effect of innovation subsidies on other dimensions of innovation in TCM firms is generally stronger than that of tax incentives, except for strategic innovation. Heterogeneity analysis indicates that the promoting effect of tax incentives is more significant in the southern TCM production region. Moreover, R&D investment mediates the relationship between innovation subsidies and innovation of TCM enterprises. Furthermore, executives with pharmaceutical backgrounds (EPB) strengthen the impact of innovation subsidies on innovation in various dimensions of TCM enterprises, while EPB only strengthens the impact of tax incentives on innovation quality and substantive innovation.

**Discussion:**

The findings provide new insights for government and TCM enterprises to promote innovation.

## 1 Introduction

Traditional Chinese medicine (TCM) is the treasure of the Chinese nation, carrying the wisdom and inheritance over thousands of years (1). For the potential side effects of some chemically synthesized drugs, people are increasingly inclined to seek safer alternatives (2). With the advantages of natural origin, minimal side effects, and low price, TCM plays an important role in health care and disease treatment (3). As a vital contributor to improving the health status of Chinese people, the government has introduced a series of policies to promote the inheritance and innovation of TCM to enhance public health (4). TCM is not only a promising field for independent innovation, but also a technological resource with original advantages in China (5). TCM enterprises are the main entities implementing TCM innovation and play a crucial role in promoting TCM innovation. The innovation of TCM enterprises is a fusion of traditional wisdom and modern technology. The TCM industry is one of the strategic emerging industries in China, and its innovation is an important way to realize the modernization, industrialization, and internationalization of TCM. Due to the high risks and costs of innovation activities, it is difficult for TCM enterprises to afford them alone (6). Thus, governments usually adopt fiscal policies to promote enterprise innovation.

Currently, fiscal policy mainly guides industrial development through government subsidies and tax incentives. The findings of existing studies on the impact of fiscal policies on enterprise innovation are inconsistent. Some scholars argue that fiscal policies can promote enterprise innovation. Gao et al. (7) found that R&D subsidies can promote firms’ exploratory innovation. In particular, under tax incentives, China’s listed integrated circuit firms can gain more technological innovation (6). Moreover, Wang et al. (8) find that government subsidies have effectively promoted innovation in new energy vehicle enterprises. Furthermore, some studies believe that other relationships between fiscal policies and enterprise innovation, such as inhibition (6, 9) and non-linearity (10, 11). Overall, existing research has established solid foundations for further discussions on the relationship between fiscal policies and innovation in TCM enterprises, but no consensus has been reached.

Due to the lack of consensus, it is difficult for the government to formulate practical policy tools and for enterprises to effectively utilize fiscal and tax resources. To explain these contradictory views, it is necessary to discuss the underlying mechanisms by which fiscal policies promote innovation in TCM enterprises and the organizational background that may affect the effectiveness of these mechanisms. This study considers research and development investment (R&D investment) as an effective channel, suggesting that fiscal policies promote the innovation output of TCM enterprises by stimulating R&D investment. Moreover, the government department is responsible for the issuance and supervision of the financial support, and the executives of the TCM companies are the decision-makers on the use of the financial support. Both of them play an important role in the implementation effect of fiscal and tax support. Upper echelons theory links executive information processing mechanisms with innovation strategies, but further explanation of the formation of executive information processing mechanisms relies on the imprinting theory (12). Imprinting theory suggests that the knowledge and skills acquired by individuals in the early stages of their education or career will shape their information processing mechanisms, persistently influencing their business decision-making behavior (13). Executives’ characteristics can affect the decision-making of enterprises (14). Executives with pharmaceutical backgrounds (EPB) may be an important factor influencing innovation in TCM enterprises. This study conjectures that EPB also affects the effectiveness of TCM enterprises in transforming fiscal resources into innovation outputs. However, there is still a lack of theoretical analysis and empirical evidence on the impact of EPB on the relationship between fiscal policy and innovation in TCM enterprises, which needs to be further verified.

Based on the above analysis, this study aims to explore the following questions: Do fiscal policies promote innovation in TCM enterprises? If so, how do R&D investment and EPB influence the relationship? To further discuss the above topics, this paper explores the impact pathways and mechanisms of fiscal policies on innovation in TCM enterprises based on data from Chinese listed TCM enterprises during 2007–2023. In particular, this paper deconstructs innovation in TCM enterprises into five dimensions, including innovation quantity (InNum), innovation quality (InQua), substantive innovation (SubIn), strategic innovation (StrIn), and inheritance innovation (InhIn). Then, this study empirically tests the effects of innovation subsidies and tax incentives on different dimensions of innovation in TCM enterprises. This study also examines (a) the heterogeneous effects across various TCM production regions, (b) whether R&D investment works as an effective pathway, and (c) the moderating role of executives with pharmaceutical backgrounds.

The contributions of this study are as follows: First, few scholars have studied the impact of fiscal policies on the innovation of TCM enterprises. This study takes TCM enterprises as the research sample, which has certain uniqueness. The TCM industry is not only a traditional industry over thousands of years, but also one of the strategic emerging industries in China. Innovation in TCM enterprises is the fusion of traditional wisdom and modern technology. Second, little literature comprehensively explores the impacts of fiscal policies on multidimensional enterprise innovation, and previous literature focuses on one or two dimensions of enterprise innovation. This paper reveals the impact of fiscal policies on multidimensional innovation in TCM enterprises, providing novel evidence for research on firm innovation. Moreover, little literature explores the role of executives with pharmaceutical backgrounds, while this study adds to existing literature. Third, this study proves that fiscal policies are of great significance to the innovation of TCM enterprises. For one thing, the findings provide a valuable reference for the government to further optimize the innovation incentive policies. For another, the results can offer new insights for TCM enterprises to better use internal experience resources and external government support to boost enterprise innovation.

The rest of this study is structured as follows: Section 2 reviews the policy background of TCM innovation and develops hypotheses. Section 3 describes samples, variables, and models. In Section 4, this study conducts empirical tests and analysis. The findings, implications, and limitations are discussed in Section 5. Section 6 concludes this study.

## 2 Theoretical background and hypotheses

### 2.1 Policy background of TCM innovation

The Chinese government has always attached great importance to TCM and formulated many policies to promote its innovation and development. The policy recognized as a landmark is the “Outline for the Modernization of TCM Development” (“Policy No. 1”) issued in 2002. Prior to this, most policies focused on TCM cultivation, with less mention of TCM innovation. Policy No. 1 proposes to build a TCM innovation system, emphasizing that the government should increase R&D subsidies and tax incentives for TCM enterprises. With this outline as a sign, the Chinese government utilized fiscal policy to promote the innovation and development of TCM enterprises into a new stage.

To maintain the continuity of policies after the expiration of partial policies in Policy No. 1, the Chinese government issued the “Outline of TCM Innovation and Development Plan” (Policy No. 2) in 2007. Policy No. 2 focuses on TCM innovation, pointing out that the use of TCM intellectual property rights should be strengthened. Entering the new era, facing insufficient inheritance and innovation of TCM, the Chinese government issued the “Opinions on Promoting the Inheritance and Innovation Development of TCM” (Policy No. 3) in 2019. Specifically, Policy No. 3 still adopts government subsidies and tax incentives as tools and utilizes fiscal funds to attract social capital to support the development of TCM enterprises. In addition to the representative policies mentioned above, there are many other policies, such as the Five-Year Plans for the pharmaceutical industry, which emphasize the use of fiscal policy to promote TCM innovation. Therefore, these fiscal policies may have a profound impact on the innovative development of TCM enterprises.

### 2.2 The impact of innovation subsidies on TCM enterprise innovation

Innovation in TCM enterprises has typical characteristics of innovation in other enterprises, such as high costs, considerable uncertainties, and significant risks. However, compared with innovation in other enterprises, innovation in TCM enterprises still has certain particularities. To begin with, the innovation of TCM enterprises reflects their innovative national characteristics. Moreover, the industrial chain of TCM enterprises is relatively long, with different innovative activities in each link from the cultivation of basic TCM plants to the production of final medicines. That is, the TCM enterprises are characterized by large-scale R&D investment, while the return period is long. Thus, it is easier for TCM enterprises to realize technological innovation through fiscal policy support.

The externality theory suggests that innovation subsidies are policy tools used by the government to support enterprises’ R&D activities and reduce market failures caused by technology spillovers. Innovation subsidy is a direct incentive policy tool, which is a free fiscal transfer provided by the government to encourage enterprises to innovate (15). As an important policy tool adopted by the government to achieve goals such as technological progress and industrial structure optimization, innovation subsidies can alleviate the resource constraints faced by TCM enterprises in carrying out innovation activities. This helps to reduce innovation costs, share R&D risks (16), increase the enthusiasm of TCM enterprises to participate in innovation, and ultimately achieve the goal of promoting the innovation performance of TCM enterprises.

Due to information asymmetry, investors are typically hesitant about projects that support innovation in TCM enterprises. Innovation subsidies reflect the importance that the government places on innovation in TCM enterprises, sending a positive signal to investors (17). This alleviates the information asymmetry and conveys that TCM enterprises supported by policies have greater market potential and investment value. Thereby, it guides investors to invest in TCM enterprises, improving investment recognition and the ability of enterprises to obtain financial resources. Thus, innovation subsidies have attracted more external investment to promote innovation in TCM enterprises. Therefore, this study proposes Hypothesis 1.

*Hypothesis 1 (H1):* Innovation subsidies have a positive effect on innovation in TCM enterprises.

### 2.3 The impact of tax incentives on TCM enterprise innovation

As an indirect incentive policy tool, tax incentives are provided by the government to reduce the tax burden for enterprises that meet certain conditions. Tax incentives mainly encourage the development of enterprises through income tax benefits, pre-tax deductions, tax rebates, and other means, which is an important measure for the government to address market failure (18, 19). Since innovation decisions of TCM enterprises depend on access to innovation resources (20), tax incentives increase their innovation funding. In addition, tax incentives also reduce innovation costs, alleviating the concerns of managers in TCM enterprises about the uncertainty of R&D activities (21). Consequently, tax incentives greatly increase the enthusiasm for TCM enterprises to pursue innovation.

Most of the current tax incentives in China have a threshold, and the government provides tax incentives to eligible TCM enterprises. TCM enterprises must apply for tax incentives based on their past innovation achievements, which has a lower distortionary effect on resource allocation and can effectively allocate innovation resources. Hence, tax incentives are considered to be a positive evaluation by the government regarding TCM enterprises’ innovation and market capabilities (19). These positive messages convey a positive signal to external investors, attracting them to increase their investment in TCM enterprises. Then more promising investment projects will be generated to promote the innovation in TCM enterprises. Accordingly, Hypothesis 2 is proposed.

*Hypothesis 2 (H2):* Tax incentives have a positive effect on innovation in TCM enterprises.

Innovation subsidies and tax incentives are common government policy tools. Due to information asymmetry, limited rationality of the government, and imperfect regulatory mechanisms, TCM enterprises may exploit loopholes to manipulate R&D activities in order to cheat innovation subsidies (22). After obtaining excess profits from innovation subsidies, TCM enterprises are more inclined to invest in non-innovative projects for profit, reducing their enthusiasm to improve profitability through long-term technological innovation. In addition, TCM enterprises may also seek rent to obtain innovation subsidies, increasing non-innovative production costs. This crowds out funds that should be used for technological innovation, resulting in the implementation of innovation subsidies being counterproductive. However, tax incentives are indirect measures with more neutral and flexible characteristics. Tax incentives avoided potential issues such as information asymmetry, fraudulent subsidies, and rent-seeking. Moreover, after obtaining tax incentives, TCM enterprises have stronger autonomy in innovation decision-making and are relatively less subject to government intervention. In addition, tax incentives generally appear in the form of laws and regulations, which is conducive to stabilizing the expectation of innovation investment of TCM enterprises and thus stimulating their long-term R&D activities. Thus, hypothesis 3 is proposed.

*Hypothesis 3 (H3):* Tax incentives are more effective in promoting innovation in TCM enterprises than innovation subsidies.

### 2.4 The mediating effect of RD

The R&D of new drugs has characteristics such as large capital demand, high innovation risk, and long investment cycles (23). The innovation achievements of TCM enterprises have strong public product attributes, while their own R&D investment is less than the socially optimal scale (24). Thus, the Chinese government supports the innovation in TCM enterprises through innovation subsidies. First, innovation subsidies can reduce the cost and risk of innovation for TCM enterprises, motivating them to increase their R&D investment (25). Second, innovation subsidies convey the government’s confidence in the innovation prospects of TCM enterprise and are more likely to attract external innovation capital (10), prompting TCM enterprises to invest more funds in innovation activities. In addition, innovation subsidies also release the signal that the innovation projects of TCM enterprises have potential market demand, which will increase their investment in R&D to seize future market share (26). R&D investment at a high level has a positive impact on innovation in TCM enterprises. Such TCM enterprises can better convert innovation subsidies into innovation outputs, thereby improving innovation in TCM enterprises. Therefore, Hypothesis 4a is proposed.

*Hypothesis 4a (H4a):* The R&D investment plays a mediating role in innovation subsidies and innovation of TCM enterprises.

Compared with innovation subsidies, tax incentives occur after the innovation activities and are considered post-incentive measures (6). On the one hand, tax incentives can reduce the tax burden of TCM enterprises and save costs for their innovative activities. That is, tax incentives reduce the uncertainty of the cash flow for TCM enterprises and have a compensatory effect on their R&D investment (27). On the other hand, tax incentives impose fewer constraints on the direction of the innovation projects implemented by enterprises (28). Hence, TCM enterprises can choose the most appropriate innovation projects according to the situation, which has a lower distortion effect on resource allocation. This will stimulate the innovation enthusiasm of TCM enterprises and thus increase their R&D investment. TCM firms with higher R&D investment levels typically have more innovative outputs. Thus, Hypothesis 4b is proposed.

*Hypothesis 4b (H4b):* The R&D investment plays a mediating role in tax incentives and innovation of TCM enterprises.

### 2.5 The moderating effect of EPB

The imprinting theory holds that the imprinted features formed by individuals during sensitive periods will have a lasting impact on their behavior (29). Even if the environment changes afterward, the features developed during these “sensitive periods” remain. Consistent with imprinting theory, existing studies have demonstrated the lasting impact of various types of characteristics developed during sensitive periods. For example, Luo et al. find that the rice culture in the birthplace of executives positively affects corporate social responsibility (30). Chen et al. believe that CEOs who have attended religious institutes are more risk-averse and will reduce their risk-taking behavior in their careers, ultimately leading to lower levels of enterprise innovation (31). He et al. explore the impact of academic experience of executives on corporate green innovation, broadening the application of imprinting theory (32). In brief, the cognitive preferences formed by executives’ experiences during sensitive periods can profoundly impact their decision-making strategies.

The TCM industry is a technology-intensive industry that has a significant impact on economic development and public health. Hence, managers of TCM enterprises require not only to be entrepreneurial, but also to have pharmaceutical backgrounds, so as to fully utilize production factors for innovation (33). First, executives with pharmaceutical backgrounds are more aware of the importance of innovation and translate this awareness into concrete actions, tending to invest more in innovation (31, 34). Second, executives with pharmaceutical backgrounds typically have a deeper understanding of products and markets (35), which enables them to more effectively formulate R&D plans and improve the success probability of innovative projects, thereby forming a competitive advantage (36). Finally, executives with pharmaceutical backgrounds also have an advantage in the implementation stage of innovation strategy. They can not only provide technical guidance, but also coordinate cross-departmental cooperation, playing the dual role of “expert + executive” (13).

There are substantial differences in the impact of executives with pharmaceutical backgrounds on innovation after obtaining fiscal and tax resources than those without pharmaceutical backgrounds. On the one hand, executives with pharmaceutical backgrounds promote TCM enterprises to carry out innovative activities in accordance with the requirements of fiscal and tax policies, so as to avoid the consumption of economic resources owing to maintaining a “special relationship” (36). On the other hand, executives with pharmaceutical backgrounds can effectively supervise the irrational use of fiscal and tax resources, alleviating the distortion of resource allocation. Overall, it is more likely for executives with pharmaceutical backgrounds to fully utilize fiscal resources for innovative activities. Hence, the following hypotheses are formulated.

*Hypothesis 5a (H5a):* Executives with pharmaceutical backgrounds strengthen the impact of innovation subsidies on innovation in TCM enterprises.

*Hypothesis 5b (H5b):* Executives with pharmaceutical backgrounds strengthen the impact of tax incentives on innovation in TCM enterprises.

**Figure 1:**
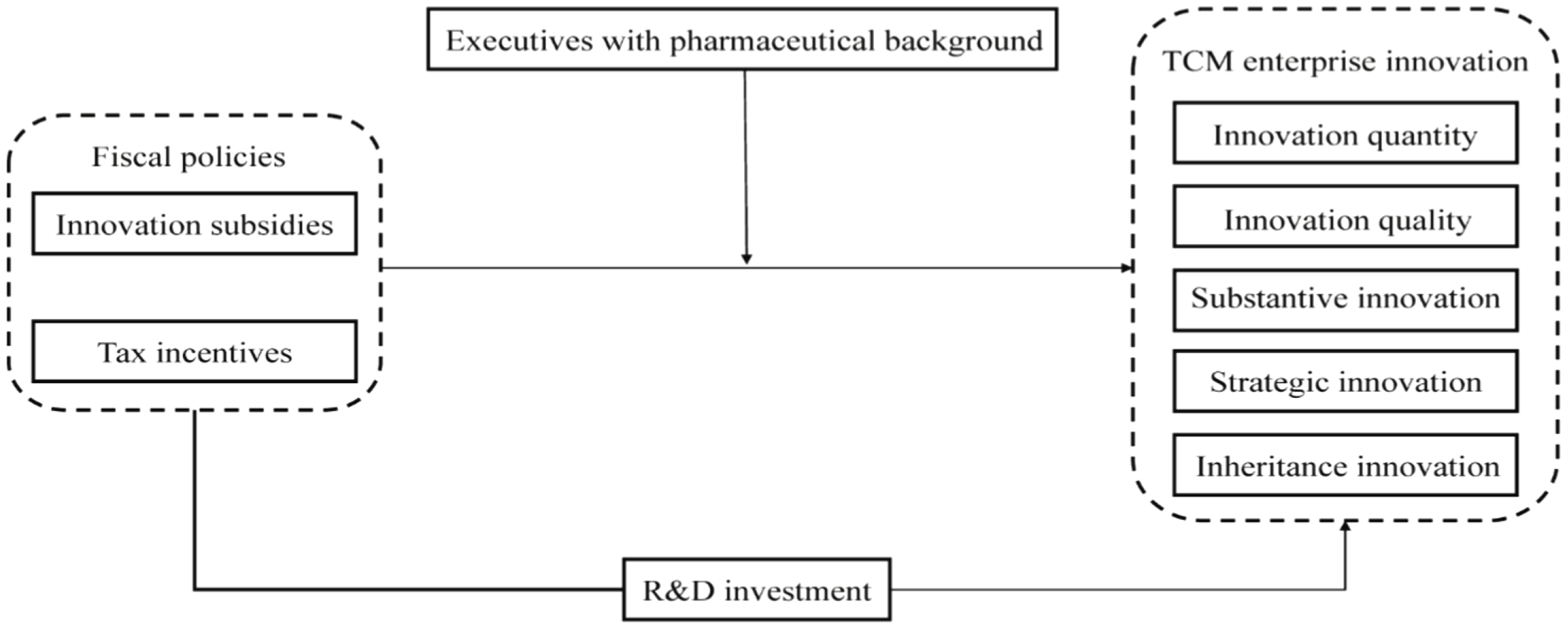
Conceptual framework.

The conceptual framework is presented in Figure 1.

## 3 Research design

### 3.1 Sample selection and data sources

According to the Shenwan Industry Classification database, this study selects Shanghai and Shenzhen A-share listed TCM enterprises as samples. The sample period in this study is from 2007 to 2023, owing to the implementation of new accounting standards and data availability. The data on TCM enterprises innovation are from the IncoPat Platform, whereas other related data is collected from the China Stock Market and Accounting Research (CSMAR) Database and annual reports. Considering data accuracy, this study removes the TCM enterprises with special treatment and missing key variables data. After data cleaning, a total of 65 listed TCM enterprises are included in this study. To avoid outliers, all continuous variables are winsorized at the quantile levels of 1 and 99%.

### 3.2 Variable selection and definition

#### 3.2.1 Explained variables

The explained variables in this study are TCM enterprises’ innovation, and existing studies typically use patents to measure firm innovation (16, 37, 38). The number of patent applications can reflect TCM enterprise innovation in a more timely manner than that of patents granted. Based on patent application indicators, this study measures TCM enterprise innovation from five aspects: innovation quantity, innovation quality, substantive innovation, strategic innovation, and inheritance innovation.

Specifically, innovation quantity is the embodiment of the total amount of enterprise innovation (39), which reflects the scale of innovation of the TCM enterprise. These innovative achievements can include, but are not limited to, new products, new technologies, new services, new methods, and so on. The increase in innovation quantity usually reflects the active degree and output capacity of TCM enterprises in innovation activities. The innovation quantity is the basis of innovation quality; without a certain amount of innovation, it is difficult to achieve a better innovation quality.

Innovation quality not only reflects the R&D strength of TCM enterprises, but also embodies the organic unity of their operational capabilities and business models (40). High-quality innovations are characterized as “transformation” and “applicability.” In this context, “transformation” means that high-quality innovation achievements that not only possess cutting-edge technology, but also have a more complex knowledge structure; “applicability” means that high-quality innovation achievements should have high commercial value and can bring direct social or economic benefits.

Substantive innovation that substantially improve the technological competitiveness of TCM enterprises is called substantive innovation (41), which aims at promoting the technological progress of TCM enterprises and gaining competitive advantages. Functional industrial policy creates a good protective environment by providing favorable conditions for the technological innovation of TCM enterprises. This will help TCM enterprises to overcome various uncertainties, giving them sufficient motivation and conditions to carry out high-quality substantive innovation.

Strategic innovation refers to the strategy of TCM enterprises to innovate in order to seek government support under the incentive policy for technological innovation, which is known as strategic innovation (42). This is due to the imperfection of the technology evaluation system and the information disclosure system and the information asymmetry of the government in screening the technological innovation capability of TCM enterprises. TCM enterprises may release false signals to the government through rent-seeking and other means to obtain R&D subsidies, leading to moral hazard and adverse selection.

Inheritance and innovation of TCM is a relationship of mutual unity, interdependence, and mutual promotion (43). The development of new TCM should emphasize the original thinking and holistic view of TCM and encourage the use of TCM research methods and modern technology to develop TCM. The government encourages the development of new TCM based on ancient classic prescriptions, experienced TCM practitioners, and TCM preparations from medical institutions, which have rich clinical experience in TCM. This requires the application of emerging science and technology to elucidate the mechanism of TCM, on this basis to promote the development and innovation of new TCM.

Therefore, research on the above five dimensions of innovation in TCM enterprises can help to better understand and promote innovation in TCM enterprises. Innovation quantity is measured by the total number of patent applications in the three categories (40); innovation quality is measured by the average number of claims of invention patents (44, 45). Following the existing studies (41, 42, 46), the numbers of invention patent and non-invention patents (i.e., utility model patents and design patents) applications are adopted to measure substantive and strategic innovation, respectively. Referring to Zhang et al. (43), by reading over 13,000 patent application texts in TCM enterprises, the number of patents citing ancient medical classics is counted to measure inheritance innovation. In terms of data processing, this paper adds one to the number of patent applications and takes the natural logarithm since the number of patents in some TCM enterprises is zero.

#### 3.2.2 Explanatory variables

The innovation incentive policy instruments typically adopted by the government include innovation subsidies and tax incentives. Thus, innovation subsidies and tax incentives are chosen as explanatory variables in this study. Referring to the measurement of Guo et al. (47), this study categorizes the details of “government subsidies” in the annual reports of TCM enterprises that contain keywords related to innovation as innovation subsidies. Specifically, this study uses keywords to determine whether they are related to innovation as follows: (1) Proper names on innovations in TCM, such as “Qhuang Project,” “inheritance and innovation,” “protection of TCM varieties,” “major science and technology projects,” “review and approval reform,” “major new drug creation,” “breakthrough therapeutic drugs,” and so on; (2) Keywords containing technological innovation, such as “R&D,” “development,” “innovation,” “science and technology,” “technology development,” “technology project funding,” “key technology application,” and so on; (3) Keywords containing science and technology support innovation, such as “Spark Program,” “Torch Program,” “863,” “Little Giant,” “high-tech enterprise,” “productivity promotion center,” “Gazelle enterprise,” “incubator,” “science and technology support program,” “standardization strategy,” and so on; (4) keywords containing innovative achievements of enterprises, such as “intellectual property,” “patents,” “copyrights,” “soft works,” and so on; and (5) Keywords containing innovative talents and technical cooperation, such as “attracting talents,” “talent storage,” “doctoral laboratory,” “elite program,” “Giant program,” “industry university research,” “foreign cooperation,” and so on. In terms of data processing, this study adds one to the innovation subsidies value and takes the natural logarithm. Following Hu et al. (38) and Wu et al. (18), this study measures tax incentives as tax rebates received by TCM enterprises. In terms of data processing, this paper adds one to the tax incentive value and takes the natural logarithm.

#### 3.2.3 Mediating variable

Existing studies suggest that fiscal and tax policies can affect firms’ innovation. Fiscal and tax policies have direct and indirect impacts on corporate innovation. Some studies have shown that fiscal and tax policies can indirectly affect enterprise innovation by stimulating R&D investment (48, 49). Hence, this study uses the natural logarithm of R&D investment as the mediating variable to explore the indirect impact of fiscal and tax policies on TCM enterprise innovation.

#### 3.2.4 Moderating variable

Executives with medical backgrounds are used as a moderating variable in this study. The information related to the career background and major background of executives can be found in the annual reports and CSMAR database. First, this study considers executives to have pharmaceutical career experience if they have worked in production, R&D, or design. Second, based on the 2024 undergraduate major catalogs in China, the majors in medical and pharmacy are selected to construct keywords for pharmaceutical majors. With this information, this study can clearly identify whether the executive has a pharmaceutical career or major background from their resume. Following Li et al. (34), the moderating variable is measured by the percentage of executives with a career background and major background in pharmaceutical areas on the top management team.

#### 3.2.5 Control variables

This study selects other factors that may affect TCM enterprise innovation as control variables. Referring to Hu et al. (38), this study controls the following variables: firm size, firm age, cash flow ratio, asset-liability ratio, equity concentration, board size, Tobin’s q ratio, and firm growth. Table 1 presents the main variables with their definitions.

**Table 1:** Variable definition table.

Variable type	Variable name	Variable symbol	Variable definition
Explained variables	Innovation quantity	InNum	Ln (the total number of patent applications +1)
	Innovation quality	InQua	Ln (average claims of invention patents +1)
	Substantive innovation	SubIn	Ln (invention patent applications +1)
	Strategic innovation	StrIn	Ln (sum of utility model and design patent applications +1)
	Inheritance Innovation	InhIn	Ln (inheritance innovation patent applications +1)
Explanatory variables	Innovation subsidies	Sub	Ln (Manually sorting out innovation subsidies disclosed in annual reports +1)
	Tax incentives	Tax	Ln (tax refunds received by TCM enterprises +1)
Mediating variable	R&D investment	RD	Ln (R&D investment of TCM enterprises +1)
Moderating variable	Executives with pharmaceutical background	EPB	Proportion of executives majoring or working in pharmaceuticals technology
Control variables	Firm size	Size	Ln (Total assets of TCM enterprises)
	Firm age	Age	Ln (Sample year- establishment year)
	Cash flow	Cash	Cash flow ratio
	Asset-liability ratio	Lev	Total liabilities/total assets
	Equity concentration	Top1	Proportion of shares held by the largest shareholder
	Board size	Board	Ln (the number of board directors)
	Tobin’s q ratio	Tobin’s q	Market value/total assets at year end
	Firm growth	Growth	(Current operating income – operating income in last year)/ operating income in last year

### 3.3 Estimation model

TCM enterprises apply innovation subsidies and tax incentives to operational activities, especially for enterprise innovation. Thus, this study explores the effects of innovation subsidies and tax incentives on the listed TCM enterprises innovation based on the following models:


(1) $$\begin{array}{l} \mathrm{Innovatio}n_{i,t+1}=\lambda_{0}+\lambda_{1}Sub_{i,t}+\lambda_{2}Tax_{i,t}+\lambda_{j}\mathrm{Contro}l_{i,t}+\\ \delta_{t}+u_{i}+\varepsilon_{i,t} \end{array}$$

In Equation 1, innovation includes five explained variables, namely innovation quantity (InNum), innovation quality (InQua), substantive innovation (SubIn), strategic innovation (StrIn), and inheritance innovation (InhIn). Sub and tax denote innovation subsidies and tax incentives, respectively. Control denotes a vector of firm-level control variables. *δ* and *u* are year and individual fixed effects, respectively. *ε* is the random disturbance term.

This study analyzes the transmission mechanism of R&D investment in the impact of fiscal policy on TCM enterprise innovation. Referring to existing studies (50), this study combines Equation 1 to construct Equations 2, 3 to examine the mediating effect of R&D investment.


(2) $$RD_{i,t}=\beta_{0}+\beta_{1}Sub_{i,t}+\beta_{2}Tax_{i,t}+\beta_{j}\mathrm{Contro}l_{i,t}+\delta_{t}+u_{i}+\varepsilon_{i,t}$$

(3) $$\begin{array}{l} \mathrm{Innovatio}n_{i,t+1}=\gamma_{0}+\gamma_{1}Sub_{i,t}+\gamma_{2}Tax_{i,t}+\gamma_{3}RD_{i,t}+\\ \gamma_{j}\mathrm{Contro}l_{i,t}+\delta_{t}+u_{i}+\varepsilon_{i,t} \end{array}$$

Based on theoretical analysis, this study constructs Equation 4 to explore the moderating effect of pharmaceutical background executives:


(4) $$\begin{array}{l} \mathrm{Innovatio}n_{i,t+1}=\alpha_{0}+\alpha_{1}Sub_{i,t}+\alpha_{2}Tax_{i,t}+\alpha_{3}{EPB}_{i,t}+\\ \alpha_{4}Sub_{i,t}\times {EPB}_{i,t}+\alpha_{5}Tax_{i,t}\times {EPB}_{i,t}+\\ \alpha_{j}\mathrm{Contro}l_{i,t}+\delta_{t}+u_{i}+\varepsilon_{i,t} \end{array}$$

## 4 Empirical results

### 4.1 Descriptive statistics analysis

Table 2 reports the descriptive statistics of the primary variables. The mean value of substantive innovation for TCM firms is 1.847, which is lower than that of strategic innovation, which is 2.089, confirming the expectation that TCM enterprises are more inclined to engage in strategic innovation. In addition, it is worth noting that the mean value of inheritance innovation is only 0.413, which initially indicates that TCM enterprises have less inheritance innovation. In terms of fiscal policy variables, the average value of innovation subsidies is higher than that of tax incentives. The average value of R&D investment of TCM enterprises is relatively high and less fluctuating, suggesting that TCM enterprises have strong initiatives to implement technological innovation. Moreover, the average proportion of executives with a pharmaceutical background is 0.301, which indicates that most executives do not have a technical background. These descriptive statistical results provide the basis for empirical investigation.

**Table 2:** Descriptive statistics.

Variable	*N*	Mean	SD	Min	Max
InNum	753	2.719	1.155	0	5.247
InQua	753	2.207	0.288	0	3.689
SubIn	753	1.847	1.078	0	4.771
StrIn	753	2.089	1.324	0	4.762
InhIn	753	0.413	0.615	0	2.996
Sub	753	15.95	1.872	0	19.92
Tax	753	8.130	7.574	0	19.52
RD	753	17.852	1.367	8.122	20.997
EPB	753	0.301	0.197	0.000	0.800
Size	753	22.03	0.992	19.32	25.09
Age	753	2.873	0.380	0.693	3.738
Lev	753	0.310	0.160	0.0410	1.893
Cash	753	0.201	0.133	0.00900	0.790
Top1	753	35.99	14.44	9.442	70.64
Board	753	1.788	0.357	0.693	2.708
Tobin’s q	753	2.492	1.623	0.697	21.30
Growth	753	14.41	61.56	−85.91	1,570

### 4.2 Baseline regression analysis

This study empirically examines the effect of fiscal policies on TCM enterprise innovation, and the results are displayed in Table 3. This paper mainly measures TCM enterprise innovation from five dimensions: innovation quantity, innovation quality, substantive innovation, strategic innovation, and inheritance innovation. The regression coefficients of innovation subsidies and tax incentives on the five dimensions of innovation mentioned above are significantly positive, which indicates that fiscal policies significantly promote TCM enterprise innovation. Due to relatively poor profitability and difficulties in external financing, TCM enterprises face severe financing constraints (51). Innovation subsidies and tax incentives alleviate the financing constraints of TCM enterprises, thereby stimulating innovation in these enterprises. Thus, H1 and H2 are supported.

**Table 3:** Baseline regression.

Variable	(1)	(2)	(3)	(4)	(5)
	InNum	InQua	SubIn	StrIn	InhIn
Sub	0.080^***^	0.023^**^	0.139^***^	0.018^***^	0.110^***^
	(7.05)	(2.16)	(6.14)	(4.16)	(3.03)
Tax	0.050^***^	0.018^***^	0.048^**^	0.100^***^	0.032^*^
	(3.79)	(2.64)	(2.56)	(3.63)	(1.74)
Size	−0.117^*^	−0.054^***^	−0.072	−0.368^***^	−0.058
	(−1.84)	(−3.08)	(−0.77)	(−4.30)	(−0.46)
Age	0.574^***^	−0.042	−2.208^***^	0.498^**^	0.232
	(3.29)	(−0.97)	(−7.06)	(2.02)	(0.41)
Cash	−0.522^***^	−0.169^**^	−0.254	0.576^**^	0.046
	(−3.25)	(−2.00)	(−1.09)	(2.54)	(0.18)
Lev	0.438^**^	−0.169^**^	0.677^**^	0.397^*^	−0.235
	(2.15)	(−2.27)	(2.27)	(1.94)	(−0.76)
Top1	0.012^***^	0.001	0.012^***^	0.007^*^	−0.009^*^
	(4.12)	(1.38)	(3.06)	(1.84)	(−1.96)
Board	0.088	−0.055^**^	−0.416^***^	0.207^**^	−0.206^*^
	(1.19)	(−2.06)	(−3.91)	(2.07)	(−1.84)
Tobin’s q	−0.028^**^	−0.002	−0.029	−0.086^***^	−0.040
	(−2.06)	(−0.26)	(−1.19)	(−4.63)	(−1.28)
Growth	−0.000	0.001	−0.002	0.000	0.001
	(−1.47)	(1.33)	(−1.45)	(1.06)	(0.93)
Constant	2.226	2.997^***^	7.000^***^	6.452^***^	−0.684
	(1.59)	(11.62)	(3.31)	(3.45)	(−0.19)
Firm	Yes	Yes	Yes	Yes	Yes
Year	Yes	Yes	Yes	Yes	Yes
N	753	753	753	753	753
*R* ^2^	0.883	0.151	0.805	0.814	0.290

^*^ *p* < 0.1, ^**^ *p* < 0.05, ^***^ *p* < 0.01; the values of *t* statistics are in parentheses.

However, there are differences in the incentives for TCM enterprise innovation between these two policy tools. Specifically, the incentive effect of the innovation subsidies on the quantity and quality of innovation in TCM enterprises is better than that of tax incentives from the value of the estimated coefficient. Since the average value of innovation subsidies received by TCM enterprises is higher than that of tax incentives, innovation subsidies can better alleviate the financing constraint dilemma and thus better encourage TCM enterprises to innovate. The impact of tax incentives on strategic innovation is greater than that of innovation subsidies. To meet the requirements of tax incentives, TCM enterprises tend to implement strategic innovation, which means applying for utility model patents with short examination times and easy authorization. In recent years, the Chinese government has introduced plenty of policies to encourage TCM inheritance and innovation (4). This paper confirms that fiscal and tax policies significantly promote the inheritance and innovation of TCM enterprises. Overall, except for strategic innovation, the incentive effect of innovation subsidies on TCM enterprise innovation appears to be generally better than that of tax incentives. Thus, H3 is rejected. The possible reasons are that, on the one hand, the current tax incentive system is not systematic, which is manifested in the unreasonable setting of the tax chain and the nonstandard implementation of tax policies. On the other hand, the scope of tax incentives is narrow, especially the tax support focused on innovative activities of TCM enterprises is insufficient, which cannot enable TCM enterprises to form stable expectations. These possible reasons make it difficult to fully exert its role in guiding the interests and sharing risks of innovation in TCM enterprises. However, as a means of direct intervention in the economy, innovation subsidy is a widely used policy tool in China, which can effectively stimulate the R&D investment of TCM enterprises and increase their proprietary knowledge accumulation and innovation output.

### 4.3 Robustness checks

#### 4.3.1 Alternative the explained variables

In the baseline regression, the innovation of TCM enterprises is measured by patent applications. Referring to Zhu et al. (17), this study adopts the number of patent authorizations to measure TCM enterprise innovation in the robustness test. The direction and significance of the coefficients on the core explanatory variables in Table 4 are similar to those in Table 3, indicating that the results are robust.

**Table 4:** Results of alternative explained variables.

Variable	(1)	(2)	(3)	(4)	(5)
	InNum	InQua	SubIn	StrIn	InhIn
Sub	0.099^***^	0.023^**^	0.061^***^	0.030^***^	0.099^**^
	(2.93)	(2.16)	(5.42)	(9.65)	(2.10)
Tax	0.050^**^	0.018^***^	0.026^***^	0.122^***^	0.035^*^
	(2.18)	(2.64)	(2.68)	(5.74)	(1.87)
Controls	Yes	Yes	Yes	Yes	Yes
Constant	−14.296^***^	2.997^***^	4.622^***^	7.744^***^	−1.819^**^
	(−16.66)	(11.62)	(3.84)	(5.27)	(−2.46)
Firm	Yes	Yes	Yes	Yes	Yes
Year	Yes	Yes	Yes	Yes	Yes
N	753	753	753	753	753
*R* ^2^	0.508	0.151	0.884	0.888	0.257

^*^ *p* < 0.1, ^**^ *p* < 0.05, ^***^ *p* < 0.01; the values of *t* statistics are in parentheses.

#### 4.3.2 Alternative the explanatory variables

This study takes the amount of innovation subsidies and tax incentives received by TCM enterprises as the explanatory variable in the baseline regression. For the robustness test, this study uses the ratio of innovation subsidies and tax incentives to business income of TCM enterprises as a substitute measurement indicator for explanatory variables (6). Replacing the measurement of explanatory variables to re-estimate, the robustness test results in Table 5 remain consistent with the baseline regression results.

**Table 5:** Results of alternative explanatory variables.

Variable	(1)	(2)	(3)	(4)	(5)
	InNum	InQua	SubIn	StrIn	InhIn
Sub	0.884^***^	0.729^**^	0.541^***^	0.227^***^	0.294^**^
	(3.74)	(2.33)	(3.90)	(3.11)	(2.42)
Tax	0.257^**^	0.123^***^	0.226^***^	0.537^*^	0.135^***^
	(2.26)	(6.78)	(3.39)	(1.71)	(4.12)
Controls	Yes	Yes	Yes	Yes	Yes
Constant	4.436^**^	−2.151^***^	−1.927^*^	3.231	−2.994^***^
	(2.46)	(−8.27)	(−1.82)	(1.30)	(−4.58)
Firm	Yes	Yes	Yes	Yes	Yes
Year	Yes	Yes	Yes	Yes	Yes
N	753	753	753	753	753
*R* ^2^	0.809	0.861	0.613	0.736	0.720

^*^ *p* < 0.1, ^**^ *p* < 0.05, ^***^ *p* < 0.01; the values of *t* statistics are in parentheses.

#### 4.3.3 Endogeneity tests

Due to potential endogeneity issues with the model, this study employs the instrumental variable approach for retesting. Referring to Zhao et al. (15), this study takes the average innovation subsidies and tax incentives obtained by other TCM enterprises in the same year as the instrumental variables. The exogenous requirement of the instrumental variable is satisfied as the fiscal and tax incentives received by the other TCM enterprises are not directly related to the innovations of the focal TCM enterprises. There is homogeneity between the incentivized TCM enterprises and other enterprises, satisfying the requirements of strong correlation. The instrumental variables selected in this study have passed the unidentifiable test and the weak instrumental variable test, verifying their effectiveness. The regression results in Table 6 confirm that the results of this study are robust.

**Table 6:** Results of the endogeneity test.

Variable	(1)	(2)	(3)	(4)	(5)
	InNum	InQua	SubIn	StrIn	InhIn
Sub	0.661***	0.242**	0.094**	0.015**	0.146***
	(11.01)	(2.21)	(2.30)	(2.30)	(4.34)
Tax	0.092**	0.031**	0.060**	0.104**	0.045**
	(2.28)	(2.27)	(2.32)	(2.36)	(2.21)
Controls	Yes	Yes	Yes	Yes	Yes
Constant	−13.513***	4.172***	−1.996	−12.215***	0.758
	(−14.07)	(4.07)	(−0.76)	(−11.74)	(0.34)
Firm	Yes	Yes	Yes	Yes	Yes
Year	Yes	Yes	Yes	Yes	Yes
LM statistic	428.211***	12.491***	595.815***	673.535***	489.405***
Wald F	447.615	26.260	335.796	209.171	449.912
Observations	753	753	753	753	753
R-squared	0.388	0.402	0.631	0.331	0.417

^*^ *p* < 0.1, ^**^ *p* < 0.05, ^***^ *p* < 0.01; the values of *t* statistics are in parentheses.

### 4.4 Heterogeneity analysis

There are differences in the level of economic development among various regions in China. When faced with incentive policies, TCM enterprises in different regions perform differently in innovation. It is important to consider the regional heterogeneity of TCM enterprises when discussing the impact of fiscal and tax policies. Thus, this study explores the impacts of fiscal and tax policies on innovation in TCM enterprises from regional heterogeneity. Specifically, according to the origin of TCM (51), this paper divides them into two categories: southern medicine production enterprises and northern medicine production enterprises.

Based on the above classification criteria, Table 7 reports the contrasting impacts of innovation subsidies and tax incentives on TCM enterprises innovation in different regions. This study finds that innovation subsidies have a positive and significant effect on the quantity and quality of TCM enterprise innovation in various regions. Moreover, tax incentives can significantly promote the innovation of TCM enterprises in southern origin, while they have little significant effect on TCM enterprise innovation in northern origin. The possible reason is that the economic development level of the southern TCM production region is higher than that of the northern TCM production region, so the tax incentive policies in that TCM production region are more numerous and powerful. From the value of average tax incentives, it shows that the average tax incentives received by enterprises in the southern TCM production region is almost $2,009,637, while that in the northern TCM production region is $492,245. That is, TCM enterprises in the northern production region receive fewer tax incentives than those in the southern production region, leading to a limited impact on innovation in TCM enterprises. Thus, the coefficients of tax incentives in the northern TCM production region, while consistent in direction with the overall regression, are not significant enough.

**Table 7:** Results of TCM production region heterogeneity test.

Variable	InNum		InQua	
	Southern medicine	Northern medicine	Southern medicine	Northern medicine
	(1)	(2)	(3)	(4)
Sub	0.041^***^	0.091^***^	0.030^**^	0.048^***^
	(3.19)	(5.67)	(2.42)	(5.63)
Tax	0.109^***^	0.011	0.013^**^	0.006
	(6.65)	(0.85)	(2.05)	(0.87)
Controls	Yes	Yes	Yes	Yes
Constant	8.758^***^	−5.196^***^	2.054^***^	2.693^***^
	(3.64)	(−3.58)	(6.69)	(9.88)
Firm	Yes	Yes	Yes	Yes
Year	Yes	Yes	Yes	Yes
N	320	433	320	433
*R* ^2^	0.690	0.282	0.458	0.269

^*^ *p* < 0.1, ^**^ *p* < 0.05, ^***^ *p* < 0.01; the values of *t* statistics are in parentheses.

### 4.5 Mechanism analysis

#### 4.5.1 The mediating role of R&D investment

To verify the possible transmission mechanism, this study chooses R&D investment as the mediating variable for testing, as shown in Table 8. The coefficient of Sub in column (1) is significantly positive, which indicates that the innovation subsidies promote R&D investment in TCM enterprises. The results in columns (2), (3), and (5) of Table 8 report that the coefficients of innovation subsidies and R&D investment are significantly positive, which suggests that R&D investment plays a partial mediating role in innovation subsidies affecting innovation quantity, innovation quality, and strategic innovation of TCM enterprises. In columns (4) and (6), since the coefficients of R&D investment are not significant, bootstrap sampling test is adopted to determine whether R&D investment plays a mediating role in the impact of innovation subsidies on substantial and inheritance innovations of TCM enterprises (34). The sampling frequency is set to 1,000 times. The results suggest that R&D investment plays a mediating role in the effect of innovation subsidies on substantive (95% Boot CI = [0.025, 0.069]) and inheritance innovation (95% Boot CI = [0.001, 0.025]). Overall, R&D investment mediates the relationship between innovation subsidies and TCM enterprise innovation. Hence, H4a is supported.

**Table 8:** Results of the mediation effects.

Variable	(1)	(2)	(3)	(4)	(5)	(6)
	RD	InNum	InQua	SubIn	StrIn	InhIn
Sub	0.035^***^	0.075^***^	0.010^***^	0.121^***^	0.015^***^	0.101^***^
	(6.22)	(4.04)	(3.37)	(6.83)	(5.95)	(3.75)
Tax	0.022	0.032^**^	0.008^***^	0.043^***^	0.083^***^	0.026^*^
	(0.96)	(2.11)	(3.42)	(4.54)	(3.22)	(1.84)
RD		0.134^***^	0.018^***^	0.045	0.123^***^	0.002
		(4.98)	(3.72)	(1.17)	(3.43)	(0.39)
Controls	Yes	Yes	Yes	Yes	Yes	Yes
Firm	Yes	Yes	Yes	Yes	Yes	Yes
Year	Yes	Yes	Yes	Yes	Yes	Yes
N	753	753	753	753	753	753
*R* ^2^	0.989	0.860	0.876	0.825	0.864	0.038

The value of *t* statistics is in parentheses, ^*^ *p* < 0.1, ^**^ *p* < 0.05, ^***^ *p* < 0.01.

In column (1) of Table 8, the coefficient of tax is insignificant, indicating that the tax incentives cannot promote R&D investment in TCM enterprises. Due to the insignificant coefficient of tax incentives, this study uses bootstrap sampling test to determine whether R&D investment plays a mediating role in the impact of tax incentives on TCM enterprise innovation. The confidence intervals of the sampling results contain 0, indicating that the mediating effect of R&D investment on tax incentives and innovation in TCM enterprises is not significant. Hence, H4b is not supported.

#### 4.5.2 The moderating role of EPB

This study examines the interaction effects between fiscal policies and pharmaceutical background executives, as shown in Table 9. The coefficients of the interaction term between innovation subsidies and pharmaceutical background executives are significantly positive in each estimation model. These results indicate that executives with pharmaceutical backgrounds strengthen the promotion of innovation subsidies to TCM enterprise innovation, and thus H5a is proven. That is, if executives have a pharmaceutical background, it will help the TCM enterprises make reasonable use of innovation subsidies and promote innovation with the advantages brought by their pharmaceutical background.

**Table 9:** Moderating effect of EPB.

Variable	(1)	(2)	(3)	(4)	(5)
	InNum	InQua	SubIn	StrIn	InhIn
Sub	0.060^***^	0.052^***^	0.039^**^	0.131^***^	0.066^**^
	(3.33)	(3.80)	(2.56)	(4.06)	(2.41)
Tax	0.029^*^	0.018^**^	0.045^***^	0.019^***^	0.025*
	(1.85)	(1.99)	(3.44)	(3.62)	(1.72)
EPB	0.133	2.221^***^	0.087	3.600^***^	0.421^***^
	(0.97)	(4.20)	(0.74)	(2.85)	(2.73)
Sub* EPB	0.214^***^	0.085^**^	0.150^**^	0.232^***^	0.332^***^
	(3.10)	(2.43)	(2.54)	(2.93)	(3.43)
Tax* EPB	0.037	0.070^***^	0.101^*^	0.283	0.023
	(1.51)	(11.01)	(1.86)	(0.14)	(1.24)
Constant	−2.646	1.935^***^	2.997^**^	−4.248^**^	−2.993^***^
	(−1.57)	(7.63)	(2.08)	(−2.53)	(−4.34)
Controls	Yes	Yes	Yes	Yes	Yes
Firm	Yes	Yes	Yes	Yes	Yes
Year	Yes	Yes	Yes	Yes	Yes
N	753	753	753	753	753
*R* ^2^	0.861	0.235	0.886	0.859	0.092

^*^ *p* < 0.1, ^**^ *p* < 0.05, ^***^ *p* < 0.01; the values of *t* statistics are in parentheses.

However, executives with pharmaceutical backgrounds weakly moderate the impact of tax incentives on TCM enterprise innovation. Specifically, as shown in Models 1, 4, and 5, these coefficients of Tax * EPB are not significant, while in Models 2 and 3, the coefficients of Tax * EPB are significantly positive. Therefore, H5b is partially supported. These results suggest that the more executives with pharmaceutical backgrounds, the more TCM enterprises tend to utilize tax incentives to enhance innovation quality and engage in substantive innovation.

## 5 Discussion

### 5.1 Discussion on the main findings

The first important finding is that greater innovation subsidies and tax incentives promote innovation in TCM enterprises, which is similar to existing studies (15, 52). Specifically, Lin et al. (15) found that fiscal subsidies and tax incentives can promote the green patent output. Kang et al. (52) also revealed that fiscal and tax policies incentivize corporate innovation. However, the above studies only measured corporate innovation in a single dimension. This study deconstructs traditional Chinese medicine enterprise innovation into five dimensions, expanding existing research. This finding is also partially inconsistent with prior research indicating that tax incentives improve innovation in the integrated circuit (IC) industry, while fiscal subsidies inhibit its technological innovation (6). A possible explanation for this difference is that industry heterogeneity leads to different effects of subsidies, with subsidies exerting a crowding-out effect in the IC industry and a crowding-in effect in the TCM industry. Moreover, some studies (28, 53) only discuss the impact of tax incentives on corporate innovation, while this study analyzes the impact of fiscal and tax policies on corporate innovation, enriching existing research. Notably, this study finds that tax incentives have a stronger incentive effect on strategic innovation than that of innovation subsidies. This finding supports the view that tax incentives tend to trigger strategic innovation strategies (42). This study further reveals that the promotion effect of innovation subsidies on various dimensional innovation of TCM enterprises is generally stronger than that of tax incentives. Moreover, this study further reveals that, except for strategic innovation, the promotion effect of innovation subsidies on other dimensional innovations of TCM enterprises is generally stronger than that of tax incentives, which complements existing research. Although TCM has a long history, chemical and biological drugs dominate the innovative pharmaceutical market. TCM enterprises have a smaller market share and weak profitability, thus facing significant financing constraints. Both innovation subsidies and tax incentives can alleviate financing constraints, while the intensity of innovation subsidies is higher than that of tax incentives in TCM enterprises, resulting in better promotion effects.

The second important finding is that innovation subsidies promote innovation in TCM enterprises through enhancing R&D investment, while the mediating effect of R&D investment on tax incentives and innovation in TCM enterprises is not significant. Similarly, Gonzalez et al. (54) argued that there is no crowding out effect between public subsidies and R&D investment and that even some small companies may not engage in R&D activities without subsidies. This is consistent with the view that innovation subsidies mitigate dual externalities by supplementing direct innovation resources, thereby guiding enterprise innovation (20, 34). One possible reason is that innovation subsidies directly supplement innovation resources compared to tax incentives. However, tax incentives are an indirect policy tool to encourage enterprise innovation, which occur to compensate firms for their R&D activities after they have done so (55). TCM enterprises face great financial difficulties, and innovation subsidies can directly incentivize them to innovate through R&D investment in a timely manner and play a leveraging effect. The effect of tax incentives on R&D investment of TCM enterprises is not obvious, which is similar to the previous study that the indirect incentive effect in developing countries is smaller than that in developed countries (56). This indicates that indirect incentives cannot induce R&D investment well for organizations facing financing constraints. Thus, the incentive effects of policy instruments are closely related to the external environment, such as the industry and country they belong to.

The third important finding is that the moderating role of EPB has a generally stronger moderating effect on innovation subsidies than tax incentives. For one thing, these findings are similar to previous literature indicating that executives with technical backgrounds contribute to the translation of subsidies into innovative performance (34). That is, Li et al. (34) found that executives with R&D experience positively moderated the relationship between subsidies and eco-innovation. Yuan et al. (33) also believed that executives with technical backgrounds can help enterprises rationally allocate R&D subsidies to promote corporate innovation. For another, this study further explores the moderating effect of EPB on tax incentives and innovation of TCM enterprises, which is rarely discussed in existing research. Interestingly, the moderating effects of EPB are more pronounced in the impact of tax incentives on innovation quality and substantial innovation of TCM enterprises. One possible explanation is that substantial innovation scores higher and plays a greater role in obtaining tax incentives (42). Thus, the more executives with pharmaceutical backgrounds, the more TCM enterprises tend to utilize tax incentives to enhance innovation quality and engage in substantive innovation. Compared with the existing literature, this study explores the moderating effect of EPB on innovation subsidies, refining the research related to executives with technical backgrounds.

### 5.2 Theoretical implications

First, this study enriches the externality theory by revealing the effects of innovation subsidies and tax incentives on various dimensions of TCM enterprise innovation. Previous studies mainly focus on the quantity and quality of innovation, with little attention paid to the antecedents of substantive innovation, strategic innovation, and inheritance innovation. Moreover, there is inconsistent evidence in the prior studies regarding the effect of innovation subsidies (9, 16) and tax incentives (6, 15, 38), and limited consideration of organizational context. This study provides new evidence for these debates and extends the application scenario of externality theory to TCM enterprises.

Second, this study enriches the imprinting theory by exploring the moderating role of executives with pharmaceutical backgrounds. Previously, although the impact of executives with R&D experience on corporate decision-making became the focus of some studies, little literature has examined the positive role of executives with pharmaceutical backgrounds on TCM enterprise innovation. The profitability of innovative activities is typically uncertain, so a major reason for the lack of innovation enthusiasm in enterprises is executives with short-sighted behavior. This study identifies the important assumption that executives with pharmaceutical backgrounds attempt to avoid short-sighted behavior and efficiently invest innovation subsidies and tax incentives into innovation activities in TCM enterprises. These results emphasize the importance of executives’ professional backgrounds in corporate innovation, thereby enriching the literature on imprinting theory and corporate innovation.

Third, this study makes contributions to enterprise innovation incentives studies by examining the impact of fiscal and tax policy on innovation in the context of the TCM industry. The TCM industry with traditional Chinese characteristics has a long history, while it is also a strategic emerging industry. Moreover, chemical drug and biological drug enterprises dominate the innovative drug market, while TCM enterprises are latecomers in this field. Thus, the findings have implications for how to strategize to incentivize innovation in latecomer firms.

### 5.3 Practical implications

This study provides implications for the government to promote innovation in TCM enterprises. First, due to the prominent promoting effect of innovation subsidies on TCM enterprises, this study suggests that policymakers increase innovation subsidies appropriately to improve innovation in TCM enterprises. Innovation subsidies provide fiscal support to alleviate the insufficient innovation investment in TCM enterprises, stimulating their innovation enthusiasm. Moreover, the government should strengthen the effectiveness evaluation and regulation power of innovation subsidies. Specifically, the government can track the progress of innovation projects of TCM enterprises in real time and dynamically arrange the scale and mode of subsequent subsidies according to the tracking results, thereby ensuring the effective use of funds.

Second, tax incentives have also demonstrated success in promoting innovation in TCM enterprises. The government should appropriately increase tax incentives to play a positive role in promoting innovation in TCM enterprises. Meanwhile, the TCM industry has a long industrial chain, and diversified tax incentives should be formulated for enterprises located in different industrial chain nodes in line with their characteristics, so as to improve the system of tax incentives. By doing so, more TCM enterprises can effectively leverage tax incentives and drive advancements in TCM enterprise innovation.

Third, the government should formulate tailored fiscal policies considering regional heterogeneity. This paper finds that the promotion effect of fiscal policies on innovation in TCM enterprises varies across production regions, which indicates that the underdeveloped northern medicine production region receives insufficient innovation resources from fiscal policies. Hence, the government should develop targeted fiscal policies for different TCM production regions and appropriately increase support for the northern TCM production region. Moreover, the central government can also utilize fiscal transfer payments to solve the funding shortage in innovation of TCM enterprises in economically underdeveloped regions.

The managerial implications for TCM enterprises are as follows: This study reveals that executives with pharmaceutical backgrounds are more capable of promoting the positive impact of fiscal policies on innovation in TCM enterprises. The pharmaceutical background of executives affects their attitudes towards innovation activities and the effectiveness of innovation subsidies and tax incentives used by TCM enterprises. Thus, TCM enterprises should value the training of executives with pharmaceutical backgrounds and give full play to their leading role in the formulation and implementation of innovation strategies to gain innovation advantages. Meanwhile, when implementing relevant fiscal policies, the background of the top management team of TCM enterprises should be fully examined.

### 5.4 Limitations and future research

This study contains several limitations. First, this study empirically analyzes the impact of fiscal policies on innovation in TCM enterprises, which is biased towards quantitative analysis. Future research could consider qualitative methods (e.g., case studies or interviews), which provide deeper understanding of the mechanisms of fiscal policies on innovation in TCM enterprises. Second, this study focuses on Chinese listed TCM enterprises. Given that the organizational structure of listed TCM firms differs from that of non-listed TCM firms, these results may not be applicable to non-listed TCM firms. Hence, future research can investigate non-listed TCM enterprises. Finally, this study mainly explores the influencing factors of innovation in TCM enterprises from the perspectives of the government and enterprise, while not incorporating the impact of other stakeholders. Future research could examine the interactions among other stakeholders (e.g., industry associations or consumers) and how they promote innovation in TCM enterprises.

## 6 Conclusion

This study examines how fiscal policies affect innovation in TCM enterprises. Innovation in TCM enterprises is deconstructed into five dimensions, including innovation quantity, innovation quality, substantive innovation, strategic innovation, and inheritance innovation. The findings are as follows: First, fiscal policies can effectively promote innovation in TCM enterprises. Specifically, the promotion effect of innovation subsidies on other dimensions of innovation in TCM enterprises is generally stronger than that of tax incentives, except for strategic innovation. Moreover, innovation subsidies significantly promote the quantity and quality of innovation in TCM enterprises in the southern and northern production regions, while tax incentives only significantly promote the quantity and quality of innovation in TCM enterprises in the southern production region. Second, R&D investment mediates the relationship between innovation subsidies and innovation in various dimensions of TCM enterprises, whereas the mediating effect of R&D investment on tax incentives and innovation in various dimensions of TCM enterprises is not significant. Third, executives with pharmaceutical backgrounds strengthen the promoting effect of innovation subsidies on innovation in various dimensions of TCM enterprises. However, executives with pharmaceutical backgrounds only strengthen the impact of tax incentives on innovation quality as well as tax incentives on substantive innovation.

## Data Availability

The original contributions presented in the study are included in the article/Supplementary material, further inquiries can be directed to the corresponding author.
