# Supplementary material for: Examining how fiscal policies influence innovation in TCM enterprises: the role of R&D investment and executives with pharmaceutical backgrounds
Source: Front Public Health. 2025 Feb 26;13:1531622. doi: 10.3389/fpubh.2025.1531622 (PMC11897503; doi:10.3389/fpubh.2025.1531622)
Supplement: Supplementary file 1 [file Table_1.docx]

**Article title: Examining how fiscal policies influence innovation in TCM enterprises: The role of R&D investment and executives with pharmaceutical backgrounds**

**Supplementary Table**: The correlation coefficient matrix of variables.

**Table S1:** The correlation coefficient matrix of variables.

| Variable | InNum | InQua | SubIn | StrIn | InhIn | Sub | Tax | RD | \| EPB \| Size \| Age \| Lev \| Cash \| Top1 \| Board \| Tobin’s q \| Growth \| \| --- \| --- \| --- \| --- \| --- \| --- \| --- \| --- \| --- \| | Size | Age | Lev | Cash | Top1 | Board | Tobin’s q | Growth |
| --- | --- | --- | --- | --- | --- | --- | --- | --- | --- | --- | --- | --- | --- | --- | --- | --- | --- | --- | --- | --- | --- | --- | --- | --- | --- | --- |
| InNum | 1 |  |  |  |  |  |  |  |  |  |  |  |  |  |  |  |  |
| InQua | 0.173*** | 1 |  |  |  |  |  |  |  |  |  |  |  |  |  |  |  |
| SubIn | 0.558*** | 0.177*** | 1 |  |  |  |  |  |  |  |  |  |  |  |  |  |  |
| StrIn | 0.428*** | 0.106*** | 0.499*** | 1 |  |  |  |  |  |  |  |  |  |  |  |  |  |
| InhIn | 0.311*** | 0.111*** | 0.397*** | 0.142*** | 1 |  |  |  |  |  |  |  |  |  |  |  |  |
| Sub | 0.389*** | 0.144*** | 0.403*** | 0.317*** | 0.167*** | 1 |  |  |  |  |  |  |  |  |  |  |  |
| Tax | 0.239*** | 0.017 | 0.157*** | 0.290*** | 0.090** | 0.414*** | 1 |  |  |  |  |  |  |  |  |  |  |
| RD | 0.489*** | 0.296*** | 0.456*** | 0.420*** | 0.181*** | 0.588*** | 0.286*** | 1 |  |  |  |  |  |  |  |  |  |
| EPB | -0.017 | 0.093** | 0.036 | -0.051 | 0.100*** | -0.039 | 0.006 | 0.092** | 1 |  |  |  |  |  |  |  |  |
| Size | 0.541*** | 0.170*** | 0.490*** | 0.493*** | 0.148*** | 0.321*** | 0.450*** | 0.590*** | -0.074* | 1 |  |  |  |  |  |  |  |
| Age | 0.215*** | 0.156*** | 0.160*** | 0.224*** | -0.025 | 0.302*** | 0.090** | 0.389*** | 0.010 | 0.448*** | 1 |  |  |  |  |  |  |
| Lev | -0.178*** | 0.013 | -0.140*** | -0.175*** | -0.010 | -0.141*** | -0.139*** | -0.124*** | 0.107*** | -0.184*** | -0.250*** | 1 |  |  |  |  |  |
| Cash | 0.156*** | -0.003 | 0.097** | 0.181*** | -0.039 | 0.202*** | 0.236*** | 0.055 | -0.091** | 0.249*** | 0.209*** | -0.379*** | 1 |  |  |  |  |
| Top1 | -0.001 | 0.091** | 0.077** | -0.065* | 0.073* | 0.150*** | -0.015 | 0.057 | 0.232*** | 0.089** | -0.227*** | 0.167*** | -0.035 | 1 |  |  |  |
| Board | 0.165*** | 0.102*** | 0.129*** | 0.170*** | 0.170*** | 0.227*** | 0.224*** | 0.184*** | 0.116*** | 0.252*** | 0.137*** | 0.008 | 0.205*** | 0.005 | 1 |  |  |
| Tobin’s q | -0.087** | 0.039 | -0.071* | -0.070* | 0.029 | -0.203*** | -0.177*** | -0.068* | 0.069* | -0.130*** | -0.102*** | 0.079** | -0.143*** | 0.111*** | -0.068* | 1 |  |
| Growth | 0.042 | -0.025 | 0.054 | 0.025 | 0.012 | -0.019 | -0.050 | -0.087** | -0.027 | -0.029 | -0.137*** | 0.021 | 0.048 | 0.047 | 0.040 | 0.078** | 1 |

Note: ^*^ *p* < 0.1, ^**^ *p* < 0.05, ^***^ *p* < 0.01.
